# Single Cell Functional Proteomics for Assessing Immune Response in Cancer Therapy: Technology, Methods, and Applications

**DOI:** 10.3389/fonc.2013.00133

**Published:** 2013-05-29

**Authors:** Chao Ma, Rong Fan, Meltem Elitas

**Affiliations:** ^1^Division of Physics, Mathematics and Astronomy, California Institute of Technology, Pasadena, CA, USA; ^2^David Geffen School of Medicine, University of California, Los Angeles, CA, USA; ^3^Department of Biomedical Engineering, Yale University, New Haven, CT, USA; ^4^Yale Comprehensive Cancer Center, New Haven, CT, USA

**Keywords:** immune function, cytokine, cancer therapy, single cell method, immune assessment, antitumor immune response

## Abstract

In the past decade, significant progresses have taken place in the field of cancer immunotherapeutics, which are being developed for most human cancers. New immunotherapeutics, such as Ipilimumab (anti-CTLA-4), have been approved for clinical treatment; cell-based immunotherapies such as adoptive cell transfer (ACT) have either passed the final stage of human studies (e.g., Sipuleucel-T) for the treatment of selected neoplastic malignancies or reached the stage of phase II/III clinical trials. Immunotherapetics has become a sophisticated field. Multimodal therapeutic regimens comprising several functional modules (up to five in the case of ACT) have been developed to provide focused therapeutic responses with improved efficacy and reduced side-effects. However, a major challenge remains: the lack of effective and clinically applicable immune assessment methods. Due to the complexity of antitumor immune responses within patients, it is difficult to provide comprehensive assessment of therapeutic efficacy and mechanism. To address this challenge, new technologies have been developed to directly profile the cellular immune functions and the functional heterogeneity. With the goal to measure the functional proteomics of single immune cells, these technologies are informative, sensitive, high-throughput, and highly multiplex. They have been used to uncover new knowledge of cellular immune functions and have been utilized for rapid, informative, and longitudinal monitoring of immune response in clinical anti-cancer treatment. In addition, new computational tools are required to integrate high-dimensional data sets generated from the comprehensive, single cell level measurements of patient’s immune responses to guide accurate and definitive diagnostic decision. These single cell immune function assessment tools will likely contribute to new understanding of therapy mechanism, pre-treatment stratification of patients, and ongoing therapeutic monitoring and assessment.

The field of targeted cancer therapeutics and immunotherapy has gone through significant maturation in recent years. For example, Ipilimumab, an antibody that blocks a T-cell function-regulating surface receptor (CTLA-4), was approved by the Food & Drug Administration (FDA) for treatment of metastatic melanoma (Hodi et al., 2010); Adoptive cell transfer (ACT) therapy that utilizes T cells expressing transgenic T cell receptor (TCR) or chimeric antigen receptor (CAR) has demonstrated high objective response rate (>40%) in Phase II clinical trials (Rosenberg, 2012). The newly approved small molecule drug, vemurafenib, or PLX 4032, that targets BRAF oncogenic mutation (V600E), has been found to induce T-cell mediated antitumor response (Sosman et al., 2012; Liu et al., 2013). Through these studies and other pre-clinical investigations, it has been increasingly recognized that immune cells play an important, yet paradoxical, role in malignancy. Cytotoxic and helper T cells, natural killer cells, and antigen presenting cells can mediate tumor destruction; whereas regulatory T cells, indoleamine-2.3-dyoxigenase (IDO) positive dendritic cells, and myeloid-derived suppressor cells (MDSCs) can protect malignancy (Hunder et al., 2008; Kantoff et al., 2010). Therefore, a deep understanding of the antitumor immune response and ways to control and maintain it are crucial for designing successful cancer therapeutics.

Immune cells execute their functions primarily through the secretion of effector or signaling proteins, jointly called cytokines. Hundreds of such molecules have been found and these cytokines can mediate a myriad of functions, from direct target killing, to self-renewal, to recruitment of other immune cell types, and to promotion or inhibition of local inflammation. Further, due to the variety of the pathogens it needs to target, cellular immunity is inherently heterogeneous at the single cell level. Individual immune cells can possess differential capacities in producing these cytokines. Therefore, a survey of immune cell function would require the development of high-throughput, highly multiplex single cell assays that can characterize the properties of single immune cells in producing multiple relevant effector cytokines, collectively called functional proteomics. An additional technical challenge is that the assays should have the capacity to relate the released proteins back to their cellular producers.

In this review, we will focus on recent progresses in the development of single cell proteomics tools, with an emphasis on those that can be used for immune diagnostics and monitoring in cancer therapeutics. These technologies are necessarily sophisticated and can generate large amounts of high-dimensional protein readouts. Therefore, advanced data modeling and analysis methods that can help interpret and visualize the readout are highly desirable. We will review some useful methods for data processing, analysis, and presentation in the second section. It is exciting that several technologies have been used to study primary human samples. Pilot studies using these technologies have provided a fresh view on the functional heterogeneity of immune cells and the dynamics of antitumor immune response. Therefore we will review some of the recent applications and propose potential roles of these technologies in cancer therapy.

## Single Cell Proteomics Technologies

Mass spectrometry in combination with liquid chromatography (MS-LC) was the first tool developed for proteomics studies. It is high-throughput and has the potential to reveal the full protein spectrum. Due to the limited amount of materials retrievable from single cells, the application of MS-LC toward single cells is challenging (Choudhary and Mann, 2010; Altelaar et al., 2013). Further, MS-LC requires input of fragmented or enzyme-digested samples and thus does not allow the recovery of viable cells for downstream usage. There have been exciting developments recently; however, the application of MS-LC in a clinical setting remains to be seen (Choudhary and Mann, 2010; Altelaar et al., 2013).

**Figure 1:**
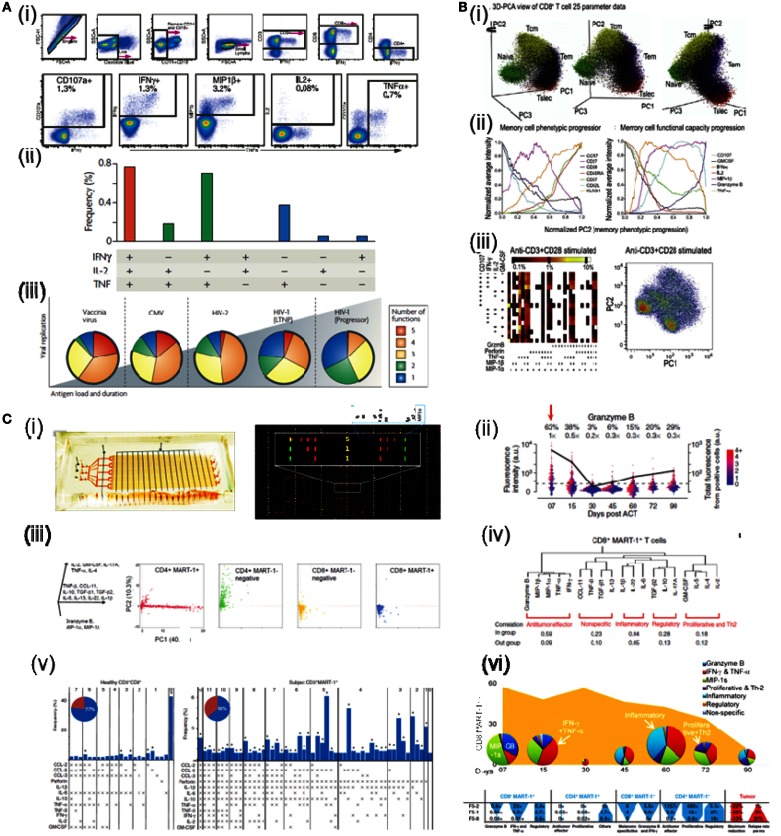
**Functional proteomics analysis by existing and emerging technologies**. **(A)** Detection of 5 concurrent T-cell functions and characterization of CD8 T-cell functionality by flow cytometry. **(i)** Gating scheme for identification of multifunctional CD8 T-cell responses. **(ii)** The T-cell response is composed of multiple functional subpopulations. Each dot denotes IFN-g, IL-2, and/or TNF-a positivity. **(iii)** The functional profile of T-cells by pie charts. For simplicity, responses are grouped by number of functions. **(B)** CD8+ T cell data measured by mass cytometry. **(i)** One data set is plotted on the first three principal component axes. **(ii)** These average expression for each phenotypic (left plot) and functional (right plot) parameters were normalized and plotted as a function of normalized PC2 values. **(iii)** Left: the combinatorial diversity of 9 T cell functions were assessed in response to anti-CD3+anti-CD28. The heat of each block represents the log scale frequency of cells displaying each combination of functional capacity. Right: psuedo-colored density-dot plots of the first two principal components are shown for cells stimulated with anti-CD3+anti-CD28. **(C)** Dynamics of antitumor immune response measured by SCBC. **(i)** The design of the single cell barcode chip (left) and sample image readout of cell cytokine production (right). **(ii)** Gated and background subtracted one-dimensional scatterplots of a representative cytokines produced by single cells at different time. **(iii)** Cytokine secretion florescence intensity data analyzed by PCA. **(iv)** Hierarchical clustering of the 19 functional cytokines produced by CD8 T cells. **(v)** Functional diversity plots for antitumor CD8 T cells. **(vi)** Time-dependent changes of T cell cytokine polyfunctional strength and comparison between three patients analyzed. (Reprint permission obtained where needed.)

Flow cytometry, invented in the 1970s, is one of the most advanced, versatile tools for studying single cells in immunology. It utilizes photon detectors to measure laser-activated fluorescence signals that are emitted from cells stained by fluorophore labeled antibodies and uses fluidics to handle the individual cells. The technology can be used to profile cell surface markers, phosphorylation during intracellular signaling and, to a limited capacity, cytokine production. With the increasing number of fluorophores available, currently 20 parameters can be measured; of them, up to 5 can be cytokines (Table 1; Figure 1A) (Perfetto et al., 2004; Betts et al., 2006). Cells can be measured at a high-throughput rate of up to 10,000/s. The potentially complicated calibration procedure to compensate the overlaps in fluorophore optical spectrum has been standardization and automated. Multiple clinical centers have established centralized flow cytometry facilities (Maecker and McCoy, 2010). A version of the flow cytometry technology, called Fluorescence Activated Cell Sorting (FACS), allows retrieving live cells with desired surface properties. Currently, as many as four cell populations can be purified in parallel. However, because of the limited multiplexity (<5), the required un-physiological blockage of protein secretion and the non-viability of cells analyzed, this technology is less optimal for measuring cytokine production.

**Table 1:** **Comparison of existing single cell technologies for profiling functional proteomics**.

Technology	Reference	Minimum sample (cells)	Current multiplexity for cytokines	Readout	Throughput	Multiplexity limitation (cytokines)	Cell recovery	Single cell level
Flow cytometry (intracellular staining)	Appay et al. (2008), Betts et al. (2006), Seder et al. (2008), Darrah et al. (2007), Bendall et al. (2012)	10^5^	3–5	Antibody staining based Fluorescence	10^4^ cells/s	<10, intracellular space, fluorophore spectrum overlapping	Yes	Yes
Mass cytometry (intracellular staining)	Bendall et al. (2011), Newell Evan et al. (2012), Bodenmiller et al. (2012), Bendall et al. (2012)	10^5^	9	Isotope	10^3^ cells/s	∼10, intracellular space, availability of isotopes	No	Yes
ELISpot	Moodie et al. (2010)	10^5^	1–3	Enzyme, fluorescence	10^6^–10^7^ cells/dish	<5	No	Quasi-single cell
Single cell barcode chip	Ma et al. (2011, 2013), Wang et al. (2012), Lu et al. (2013), Ma et al. (2012a,b)	10^4^	20	Fluorescence	10^3^–10^4^ cells/chip	100–1,000	No	Yes
Micro-engraving	Han et al. (2012), Varadarajan et al. (2011, 2012), Yamanaka et al. (2013)	10^4^–10^5^	3	Fluorescence	10^3^–10^5^ cells/chip	<5	Yes	Yes

One recent technical breakthrough along the direction of flow cytometry is mass cytometry, also known as cytometry by time-of-flight (cyTOF) (Bendall et al., 2011). The technology is based on the detection of isotopes that do not naturally exist in biologically samples. Cells are stained by isotope-labeled antibodies and are then “evaporated” into clouds of molecules in the machine; thereby the isotope labeling is detected. The application of this technology in immunology was first reported in 2011 (Bendall et al., 2011). With proper combinatorial barcoding, the technology has been showed to detect 30 surface markers and 9 cytokines simultaneously (Table 1; Figure 1B) (Bodenmiller et al., 2012). Unlike flow cytometry, whose multiplexity is limited by the overlaps in fluorophore spectrum, mass cytometry can potentially detect a huge number of markers simultaneously (Bendall et al., 2012). Currently, the technology is limited by the comparatively low-throughput rate and the low fraction of sample analyzable; however, it is expected to improve (Bendall et al., 2012). Because cells are “evaporated” during the assay, cells cannot be retrieved for downstream analysis. Thanks to many shared components and established experience available from flow cytometry, this technology grows very fast. It has been used to study the hierarchy of hematopoietic stem cell differentiation, the natural killer cell intracellular signaling and the T cell functional heterogeneity (Bendall et al., 2011; Bodenmiller et al., 2012; Newell Evan et al., 2012), as we will review later.

The enzyme-linked immunosorbent spot (ELISpot) or fluorospot assay is a widely used quasi-single cell technique (Moodie et al., 2010). In the assay, cells are cultured on a petri dish that is pre-coated with cytokine-specific antibodies. Cytokines released from individual cells are captured by surrounding antibodies. Subsequently, these captured cytokines are detected by applying secondary antibodies and fluorophore labels or through enzymatic reaction. After the assay, the number of spots on the petri dish, each relating to a cytokine-producing cell, can be enumerated. ELISpot can achieve a high sensitivity (<0.1%) and allows the detection of one to three cytokines at the same time (Table 1). Because single cells are not separated during the measurement, the protein level cannot be quantitated and individual cells cannot be distinguished when cells are too close together.

Recent developments in microfluidics have revolutionized the traditional ELISpot assay. These microchip-based technologies utilize arrays of highly miniaturized nano- to pico-liter volume micro-chambers to achieve ultra-sensitive protein measurement and the separation of single cells. Because single cells are separated in different micro-chambers, their protein levels can be quantitated in parallel. About 1,000–10,000s micro-chambers can be integrated into one microchip, to achieve high-throughput measurements. The amenability of these technologies to integrate with upstream cell purification and on-chip optical imaging further enhances their utility. Moreover, microchips are highly portable, low-cost, and are sample-efficient.

One version of these microchips is called the Single Cell Barcode Chip (SCBC) (Ma et al., 2011). It couples a microfluidics-generated antibody microarray substrate with a microfluidics chip containing a large array of micro-chambers. The antibody microarray serves to detect cytokines secreted and the microchip is designed to fit a full panel of antibodies in each micro-chamber. During the assay, single cells are loaded into these 100-pl size micro-chambers. Because of a 1-million fold miniaturization, the microchip can achieve ultra-sensitivity down to 100 molecules and only requires 10,000 cells as starting material. Currently, more than 20 proteins can be measured simultaneously from 5 to 10 thousand micro-chambers (Table 1; Figure 1C) (Ma et al., 2011, 2012a,b, 2013; Wang et al., 2012; Lu et al., 2013). The technology has been applied across many fields, including studying adaptive, innate immune cells, hematopoietic stem cells, and intracellular signaling in malignancy (Ma et al., 2011, 2012a,b, 2013; Wang et al., 2012). In particular, this technology has been used to study the functional heterogeneity of human T cells and clinical immune responses in an ACT immunotherapy to metastatic melanoma (Ma et al., 2011, 2013).

Another version of the microchips employs the micro-engraving technique to fabricate micro-chambers (Varadarajan et al., 2011; Yamanaka et al., 2013). In this technology, hundreds of thousands nano-liter sized micro-chambers can be integrated into one chip, wherein up to three types of cytokines can be measured by antibody on the substrate (Table 1). At the same time, cells can be stained by three colors. Immune cell – target cell interaction can be measured by on-chip imaging and temporal cytokine production profile can be acquired by periodically switching the antibody substrates (Varadarajan et al., 2011; Han et al., 2012). This technology also has the capacity to retrieve viable individual cells with desirable properties from the microchip, as has been showed in the case of T cell cloning (Varadarajan et al., 2012). Moreover, it has also been used to show the discordant cytokine production dynamics of human T cells (Han et al., 2012; Yamanaka et al., 2013).

The features of the technologies reviewed are summarized in Table 1.

## Analysis Methods

The massive, high-dimensional data generated by cytometry and microchips has spurred the development of computational analysis methods.

The cytokine signals are normally measured in fluorescence intensity. To compare data acquired from different samples and from different experiments, the background level specific for each protein needs to be identified and subtracted. One logical way to characterize cells is to divide them into cytokine-producing and non-producing fractions by a gate in fluorescence level. Then, one can focus on properties of the cytokine-producing fraction by calculating their relative abundance as well as their cytokine production intensity.

For flow cytometry, commercial software, such as BD Diva and FlowJo, has been developed that can provide simple data analysis capacity. Such software can generate one-dimensional distribution plots and density-based two-dimensional plots and allows the user to manually gate out desirable cell subpopulations (see example in Figure 1Ai). However, manual gating is subjective and laborious, and can generate inconsistent results when a large number of proteins and samples are analyzed.

An alternative approach to detect background and determine gate is to utilize computational algorithms to fit the density distribution. Finite mixture models and their variants are commonly used (Reynolds and Rose, 1995). Some models take into account the skewness and kurtosis of the measured distribution and could generate good result in many cases (Pyne et al., 2009). In parallel, non-parametric methods have been developed to extract features of the distribution (Walther et al., 2009; Ma et al., 2013).

The single cell functional heterogeneity can be characterized after cytokine-producing and non-producing cells are identified. For example, cells can be grouped into subpopulations that produce different number of cytokines and the relative abundance of each group can be showed in a pie graph (Betts et al., 2006; Seder et al., 2008; Ma et al., 2012a,b) (Figures 1Aii,iii). Such a plot reflects the functional distribution. Different pie charts can be compared for statistically significant differences (Betts et al., 2006; Seder et al., 2008). A more thorough way to look at this functional heterogeneity would be to further subdivide cell population into subpopulations producing different combinations of cytokines. Then, the distribution can be showed as a bar group with an accompanying matrix denoting the function combinations (Figures 1Aii, Bi–iii, Ci–v) (Betts et al., 2006; Ma et al., 2011; Newell Evan et al., 2012). This type of representation is informative and has been used to show the profound functional heterogeneity existed in T cell populations actively attacking tumor, comparing to that of resting T cells (Ma et al., 2011). Furthermore, statistical indicators summarizing the functional heterogeneity can also be defined based on this information (Figure 1Cvi) (Seder et al., 2008; Ma et al., 2013).

Since the ultimate goal of gating is to identify biologically significant cell subpopulations based on the type and level of cytokines produced, computational methods have been developed that directly model the distribution of the multi-dimensional cytokine data. Such methods utilize different versions of clustering method, such as k-means clustering, hierarchical clustering, and their variants (Figure 1Civ) (Johnson and Wichern, 2007; Aghaeepour et al., 2011). The basic idea is to group the data points by certain measure of point–point distance in the high-dimensional space representing the cytokines measured. One of the challenges to utilize clustering methods is to pre-define the number of clusters exist. Most of time, such information is not known beforehand, therefore additional indicators and trial-and-error iterations are necessary. The gating methods and grouping methods have provided very promising results in many cases; however, due to the often-existed complexity and irregularity of cell population, none of these methods has showed widespread successes (Zare et al., 2010; Aghaeepour et al., 2011).

High-dimensional analysis is especially susceptible to multiple data defects, an effect called curse of dimensionality (Johnson and Wichern, 2007). First, the amount of data required to allow meaningful analysis increases exponentially with the number of proteins measured. Second, spurious correlation is more likely to happen in high-dimensional data and the measure of distance used for clustering analysis is prone to be invalid. Lastly, statistical tests need to be redesigned when repetitively used for high-dimensional data, as true type I error can be much larger than expected.

To address these challenges, methods have been developed to “concentrate” the information by reducing the dimensionality. Such an approach is also biologically sound: due to the interrelating nature of gene transcription and protein expression, protein signals are normally correlated with each other. Therefore, only a small number of truly independent variables or “degrees of freedom” exist that define the biological process. In this regard, principal component analysis (PCA) and its variants are powerful resorts (Johnson and Wichern, 2007; Ma et al., 2012a,b, 2013; Newell Evan et al., 2012) (Figures 1Bi,ii, Ciii). When data is meaningful and the analysis is applied correctly, different components representing different aspects of biological information can are discovered. At the same time the noise is reduced. Other recent development in this direction utilized minimum spanning tree and clustering methods to characterize and display the high-dimensional data on a two-dimensional plane and provided a revealing illustration of hematopoietic stem cell differentiation (Bendall et al., 2011).

## Clinical Applications and Future Directions

The application of these new technologies has greatly advanced our understanding of functional heterogeneity within immune cells. Initial studies (Ma et al., 2011; Newell Evan et al., 2012) on human T cells showed the existence of profound functional heterogeneity within a population of genetically and phenotypically similar T cells and demonstrated that the level of functional heterogeneity reflects the functional activity of T cells (Ma et al., 2011, 2012a,b, 2013). The functional heterogeneity has also been showed to be highly focused and the distribution of functional subsets is significantly different from a random distribution (Ma et al., 2011; Newell Evan et al., 2012). Thus, the functional heterogeneity contains valuable biological information, rather than random biological noise.

A new insight emerges from flow cytometry and microchip analysis is that a fraction of cells, called the polyfunctional cells, can simultaneously secrete a large number of cytokines. They also secreted each of these cytokines in large amounts (Betts et al., 2006; Darrah et al., 2007; Seder et al., 2008; Ma et al., 2011, 2013). Thus, they produced a predominant amount of cytokine in an immune response (Ma et al., 2013). One explanation of this phenomenon is that the cytokine functions are coordinated at the level of single cells and new parameters have been defined to summarize this information of polyfunctionality (Figure 1Cvi) (Darrah et al., 2007; Seder et al., 2008; Ma et al., 2013). These parameters have been found to correlate with the quality of T response in human and animal models (Darrah et al., 2007; Seder et al., 2008; Ma et al., 2013). For example, an index, named polyfunctional strength index (pSI), is developed to summarize the joint functional intensity from polyfunctional T cells and its distribution among cytokines (Ma et al., 2013). It is used in a recent study that monitored the temporal changes of antitumor T cells retrieved from metastatic melanoma patients participating in a transgenic TCR ACT immunotherapy. By comparing the changes in the frequency, phenotype, and polyfunctionality (summarized by pSI) of these T cells, the study showed that only the functional changes are highly distinguishable between patients and that the changes correlated with the clinical outcome (Ma et al., 2013) (Figure 1Cvi).

These studies demonstrated the importance to understand the functional heterogeneity of immune cells and its preliminary value in clinical diagnostics and monitoring. Because both the cellular immunity and tumor are heterogeneous at the single cell level, successful cancer therapeutic scheme is necessarily personalized. Therefore, personalized diagnostic and monitoring tools, such as the single cell functional analysis, are highly desirable and can be a integrative component in the cancer therapeutics. By understanding the functional characteristics of their immune cells, patients can be stratified pre-treatment for the best available treatment and their immune response can be monitored during the therapy so that further intervention can be applied timely. The massive information acquired are also valuable feedbacks to guide further improvements of cancer therapy.

## Author Contribution

Chao Ma conceived and composed the paper. All authors reviewed the manuscript.

## Conflict of Interest Statement

The authors declare that the research was conducted in the absence of any commercial or financial relationships that could be construed as a potential conflict of interest.
